# Dual-gRNA CRISPR/Cas9 Deletion of CsDMR6 in Sweet Orange Supported by Improved In Vitro Regeneration

**DOI:** 10.3390/plants15172664

**Published:** 2026-08-31

**Authors:** Sandra Sopalda, Ricardo Vergara, Marisol Muñoz, Carlos Aguirre, Carlos Muñoz, Humberto Prieto

**Affiliations:** 1 Master’s Program in Agricultural Sciences, Faculty of Agricultural Sciences, University of Chile, Santiago 8820808, Chile; sandra.sopalda@gmail.com; 2 Biotechnology Laboratory, Institute of Agricultural Research–La Platina, Santiago 8831314, Chile; ricardo.a.vergara.g@gmail.com (R.V.); mmunoz@inia.cl (M.M.); caguirre.d@gmail.com (C.A.); 3 Plant Breeding Laboratory, Faculty of Agricultural Sciences, University of Chile, Santiago 8820808, Chile

**Keywords:** CRISPR/Cas9, *CitrusS sinensis*, *CsDMR6*, geminivirus vector, Huanglongbing, in vitro regeneration, susceptibility gene

## Abstract

Huanglongbing (HLB), caused by *Candidatus Liberibacter* spp., remains the most destructive disease affecting citrus worldwide. To support host-directed genome-editing strategies aimed at reducing susceptibility, we optimized key regeneration steps in *Citrus sinensis* and validated a dual-gRNA CRISPR/Cas9 approach targeting the susceptibility gene *CsDMR6*. Juvenile explants of ‘Valencia’ and hybrid genotypes (CsH1–CsH3) were successfully established in vitro, and shoot elongation was markedly improved by supplementing Citrus Shoot Multiplication (CiSM) medium with 1 mg L^−1^ GA3. Callus induction was most efficient in Citrus Callus Induction (CiCM) medium under dark conditions, while a 48 h NAA pulse (100 µM) significantly enhanced rooting, increasing efficiencies to 37.1% in ‘Valencia’ and 52.9% in CsH1. Two guide RNAs targeting conserved regions of *CsDMR6* were designed and shown to be identical across all evaluated genotypes. The dual-gRNA cassette was assembled into a CRISPR/Cas9 geminivirus-based vector and transiently delivered into sweet orange leaf tissue via *Agrobacterium*. GFP fluorescence verified construct expression, and PCR amplification across the target region produced a diagnostic ~447 bp fragment corresponding to the expected ~5.8 kb deletion. Sanger sequencing confirmed precise junction formation between the two cut sites. These results demonstrate efficient large-fragment deletion of *CsDMR6* in sweet orange and establish an experimentally validated, genotype-compatible regeneration and editing platform. This study provides a transient validation of the dual-gRNA system and establishes the technical foundation required for future stable, non-transgenic edited lines. Together, these advances support the downstream functional evaluation of *CsDMR6* loss-of-function alleles under HLB pressure.

## 1. Introduction

Huanglongbing (HLB), or citrus greening, is the most destructive disease affecting citrus production worldwide [1]. The disease is associated with the phloem-restricted bacteria *Candidatus Liberibacter* spp., primarily *Ca. L. asiaticus*, transmitted by the psyllid vectors *Diaphorina citri* and *Trioza erytreae* [2]. Infected trees exhibit leaf chlorosis, blotchy mottle, phloem plugging, and progressive canopy decline caused by callose deposition and protein accumulation, ultimately leading to tree death [3]. Since no curative treatment is available, and no commercially resistant sweet orange cultivars exist, the development of durable host-based resistance has become a central priority for sustainable citrus production [4].

Additionally, conventional breeding for HLB resistance is severely limited by the long juvenile phase, high heterozygosity, and recalcitrance of citrus, underscoring the need for biotechnological approaches capable of directly modifying host susceptibility. These limitations highlight the importance of strategies that can generate resistance without relying on lengthy breeding cycles.

One promising strategy involves modifying host susceptibility (S) genes. These genes are exploited by pathogens to facilitate colonization, and their inactivation can reduce disease severity by generating a loss-of-susceptibility phenotype [5]. Among the candidate S genes in citrus, *Downy Mildew Resistance 6* (*DMR6*) has emerged as a compelling target. *DMR6* encodes a 2-oxoglutarate [Fe (II)]-dependent oxygenase that functions as a negative regulator of plant immunity [6]. Transcriptomic studies have shown that *CsDMR6* is strongly induced during HLB infection in susceptible citrus genotypes [7], suggesting that targeted disruption of *CsDMR6* may reduce host vulnerability.

Although the precise role of DMR6 in citrus immunity remains to be fully validated, its induction during HLB infection and its established function in other species support its consideration as a strategic susceptibility gene. Recent comparative transcriptomic analyses further support the relevance of targeting susceptibility pathways in citrus. Wang et al. [8] reported that HLB-tolerant grapefruit hybrids activate broad basal defense programs, whereas susceptible genotypes exhibit suppression of multiple immunity-associated routes. This contrasting immune configuration aligns with the strong induction of *CsDMR6* during HLB infection and reinforces its role as a strategic susceptibility gene that, when disrupted, may hypothetically enhance host resilience. Importantly, editing *CsDMR6* has already been shown to confer resistance to citrus canker caused by *Xanthomonas citri* [9], validating its role as a functional susceptibility gene in citrus. Nevertheless, the extent to which *CsDMR6* disruption may influence HLB tolerance remains unknown and requires functional evaluation in edited plants.

Recent advances further highlight the growing interest in host-directed editing for HLB management. Targeted mutagenesis of callose synthase 7 (*CalS7*) in Indonesian mandarin genotypes is being explored as a strategy to mitigate HLB [10], while evolutionary frameworks suggest that modifying conserved susceptibility pathways may confer durable resistance to *Ca. Liberibacter* [11]. In parallel, modern reviews emphasize the rapid expansion of genome editing in citrus and the need for efficient platforms to overcome the species’ recalcitrance [12]. Citrus regeneration remains a major bottleneck for genome editing, as shoot elongation, rooting, and genotype-dependent responses often limit the recovery of edited plants. This gap highlights the need for experimentally validated regeneration workflows that support CRISPR-based editing. Developing such workflows is essential for enabling reliable recovery of edited citrus lines.

CRISPR/Cas9 genome editing provides a precise and efficient approach for generating loss-of-function alleles in S genes [13]. Dual-guide RNA (dual-gRNA) architectures are particularly advantageous because they generate large, easily detectable deletions rather than small indels, thereby simplifying molecular screening and increasing the likelihood of complete gene inactivation [14,15,16,17]. However, the application of genome editing in citrus remains constrained by the species’ well-known recalcitrance to in vitro regeneration [12]. Although shoot induction can be achieved, consistent elongation and rooting, especially in commercial hybrids, remain major technical bottlenecks [18]. Thus, establishing a robust regeneration platform is a prerequisite for enabling efficient CRISPR/Cas9 editing in sweet orange. Such a platform is also necessary to support downstream recovery of stable edited plants.

Here, we refined key regeneration steps in *C. sinensis* and validated a dual-gRNA CRISPR/Cas9 strategy to generate a large deletion in *CsDMR6*. We hypothesized that dual-gRNA targeting of *CsDMR6* would generate a complete loss-of-function allele through a large deletion, and that optimized regeneration conditions would establish a viable platform for recovering these edited lines. Sequence conservation analyses across the genotypes confirmed complete identity at both gRNA sites, reinforcing the suitability of *CsDMR6* as a broadly applicable susceptibility gene target. Together, these results establish an experimentally supported platform for future recovery of stable edited lines and their functional evaluation under HLB pressure. This work addresses a critical gap in citrus biotechnology by integrating regeneration optimization with dual-gRNA editing, providing a foundation for future development of non-transgenic, loss-of-susceptibility citrus germplasm. Overall, the study contributes both a practical regeneration workflow and a validated editing strategy that together enable progress toward host-directed HLB resistance.

## 2. Results

### 2.1. Optimization of Aseptic Establishment and Shoot Morphogenesis

Aseptic establishment was more efficient in seed-derived embryos than in juvenile internodal segments. Embryos from ‘Valencia’ and the hybrid genotypes CsH1, CsH2, and CsH3 showed high survival after sterilization, whereas internodal segments exhibited lower viability due to their greater exposure to environmental contaminants and sensitivity to surface disinfectants. A total of 12 explants of ‘Valencia’, 12 of CsH1, 16 of CsH2, and 24 of CsH3 were introduced into culture, and explants were subcultured every 30 days. Seed-derived embryos germinated within ~30 days, while internodal segments produced axillary shoots after ~45 days.

CiSM1.0 and CiSM1.25 supported consistent shoot proliferation in all genotypes. However, shoots grown on CiSM1.0 displayed a compact phenotype characterized by dense leaf clusters and minimal internode elongation. This compact phenotype became evident between 20 and 30 days of culture and was consistently observed across all genotypes. CiSM1.25 resulted in high contamination rates and weak shoot development, while CiSM2.1+KIN produced thin, unstable shoots with irregular elongation, leading to the discontinuation of both formulations. These outcomes explain why only CiSM1.0 was selected for further optimization.

**Figure 1:**
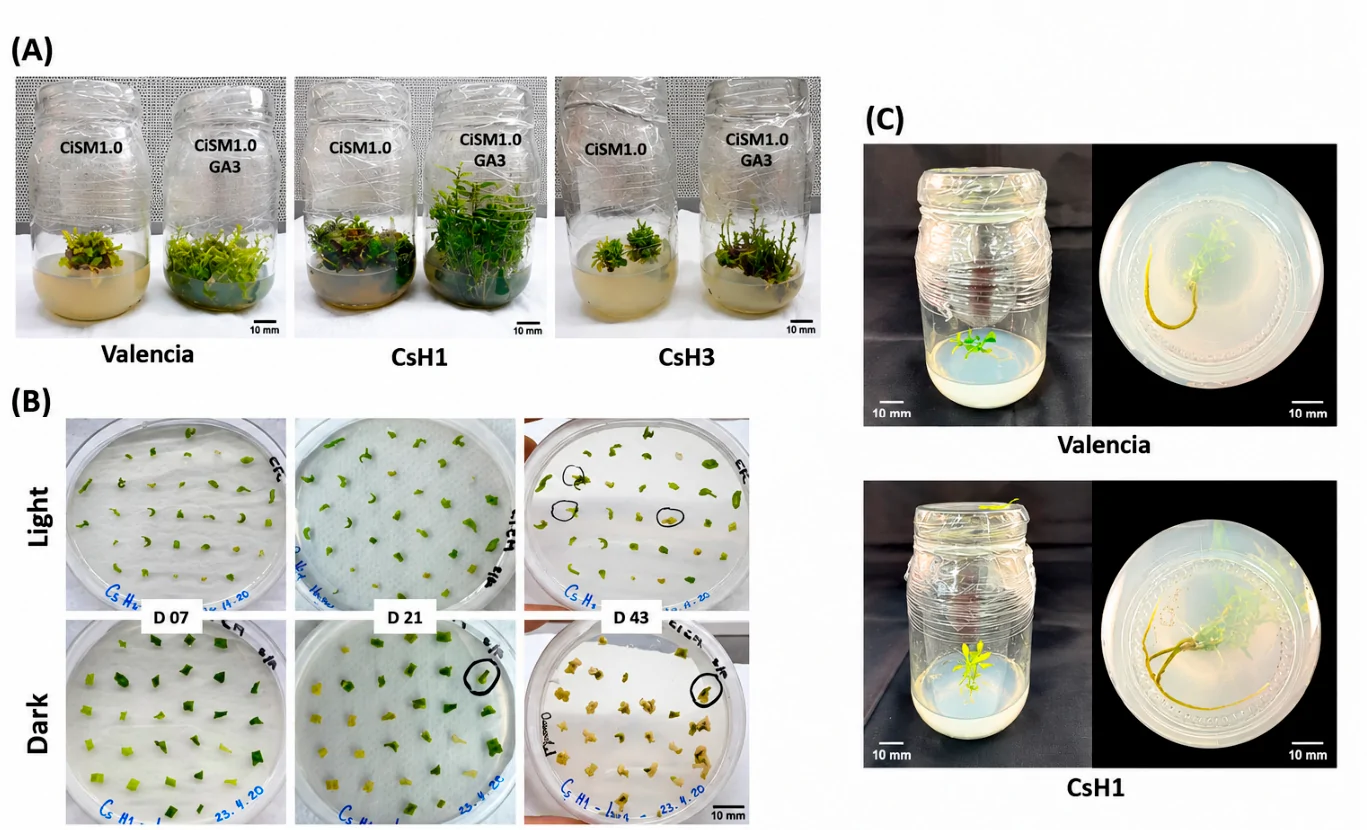
Optimized regeneration platform supporting gene editing in *Citrus sinensis*. (**A**) Supplementation of CiSM1.0 medium with 1 mg L^−1^ GA3 promotes internode elongation and improves shoot morphology, generating explants suitable for downstream transformation. (**B**) Callus induction is significantly enhanced in CiCM medium under dark conditions, producing friable, cream-colored calli at a higher frequency than under photoperiod conditions. (**C**) A 48 h NAA pulse (100 µM) markedly increases rooting efficiency in recalcitrant hybrid genotypes, thereby enabling reliable shoot recovery for gene-editing workflows. Circled areas indicate early callus formation observed in both light and dark conditions, representing a normal tissue response (**B**).

Supplementation with 1 mg L^−1^ GA3 resolved this limitation by promoting internode extension and producing elongated shoots suitable for subculturing and rooting (Figure 1A). GA3-induced elongation became visible after 10–14 days. While ‘Valencia’, CsH1, and CsH2 responded uniformly, CsH3 exhibited excessive elongation with long internodes and reduced leaf development, indicative of GA3 overstimulation. Improved gas exchange using ventilated culture jars further enhanced shoot vigor. Genotypic differences were evident in regeneration performance, with ‘Valencia’ showing higher regeneration frequencies and shoot production than CsH1 (Table 1). Regeneration frequency is defined as the percentage of explants producing shoots. CsH2 and CsH3 also regenerated, though at lower frequencies and with slower shoot development, consistent with their behavior during aseptic establishment.

### 2.2. Optimization of Rhizogenesis for Plant Recovery

Because shoot elongation is a prerequisite for downstream experimentation, rooting performance was evaluated immediately after shoot regeneration. Rooting responses varied significantly among genotypes. In hormone-free MS½ medium, ‘Valencia’ exhibited a baseline rooting frequency of 30.3%, whereas CsH1 did not root at all under these conditions. To quantify genotype-specific rooting behavior, a total of 10–15 elongated shoots per genotype were evaluated, derived from the regeneration experiments described in Section 2.1. Larger shoots (>2 cm) rooted more consistently than smaller shoots, which frequently produced callus instead of roots. Initial root primordia became visible after approximately 20 days.

The application of a 48 h NAA pulse (100 µM) markedly improved rooting across genotypes (Figure 1C). After the pulse, ‘Valencia’ reached 37.1% rooting, and CsH1 reached 52.9%. Hybrid genotypes CsH2 and CsH3 also responded positively to the auxin pulse, although with slower root emergence and lower overall rooting frequencies, consistent with their reduced vigor during shoot morphogenesis. Shoots with larger leaf surfaces responded more favorably, achieving 52.9% rooting compared to 35.3% in smaller shoots. Following root formation, plantlets were transferred to soil and acclimatized under high humidity for 10–14 days, achieving >80% survival across genotypes.

These results confirm that the auxin pulse is essential for reliable rhizogenesis in recalcitrant hybrid genotypes and provides a functional bridge between regeneration and gene-editing workflows. Together, these genotype-specific responses highlight the importance of shoot quality and controlled auxin exposure for supporting downstream recovery of edited citrus lines.

### 2.3. Molecular Characterization of the CsDMR6 Target and Vector Assembly

**Figure 2:**
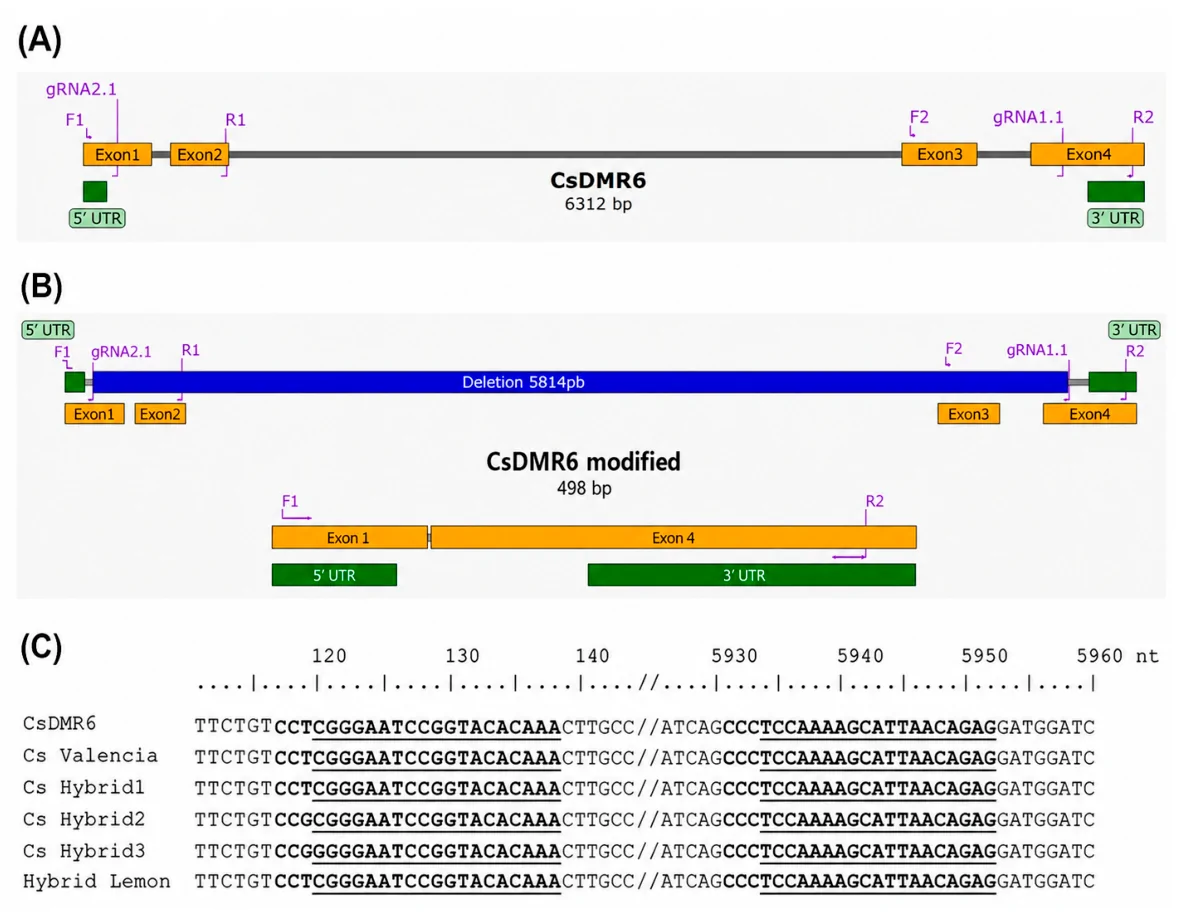
Dual-gRNA design targeting the *CsDMR6* locus and conservation of editing sites across citrus genotypes. (**A**) Gene structure of *CsDMR6* showing four exons and the positions of gRNA2.1 (Exon 1) and gRNA1.1 (Exon 4), designed to generate a ~5.8 kb deletion upon simultaneous Cas9 cleavage. (**B**) Schematic representation of the predicted deletion and diagnostic PCR strategy using flanking primers CsDMR6-F1/CsDMR6-R2. (**C**) Sequence alignment of the target regions across sweet orange and hybrid citrus genotypes confirms complete conservation of both gRNA sites (in bold and underlined) and PAM motifs (in bold), supporting the broad applicability of the editing strategy.

The *CsDMR6* locus spans approximately 6.3 kb and contains four exons (Figure 2A). The dual-gRNA design targeted conserved regions in Exon 1 (gRNA2.1) and Exon 4 (gRNA1.1), with a predicted deletion of 5814 bp (Figure 2A,B). Sequencing of the target regions in ‘Valencia’, CsH1, CsH2, CsH3, and a hybrid lemon genotype revealed 100% conservation of both gRNA sites and PAM sequences (Figure 2C). Multiple sequence alignments confirmed complete identity at both gRNA loci and their adjacent PAMs across all genotypes, supporting the broad applicability of the dual-gRNA design for citrus editing. The dual-gRNA cassette was successfully assembled into the pVK:CsDMR6A vector, producing a diagnostic 926 bp amplicon, and Sanger sequencing confirmed correct assembly. This 926 bp fragment corresponds to the assembled dual-gRNA module within the geminiviral backbone and serves as a molecular indicator of correct construct integration prior to transformation.

### 2.4. Transient Expression and Evidence of Targeted Deletion

**Figure 3:**
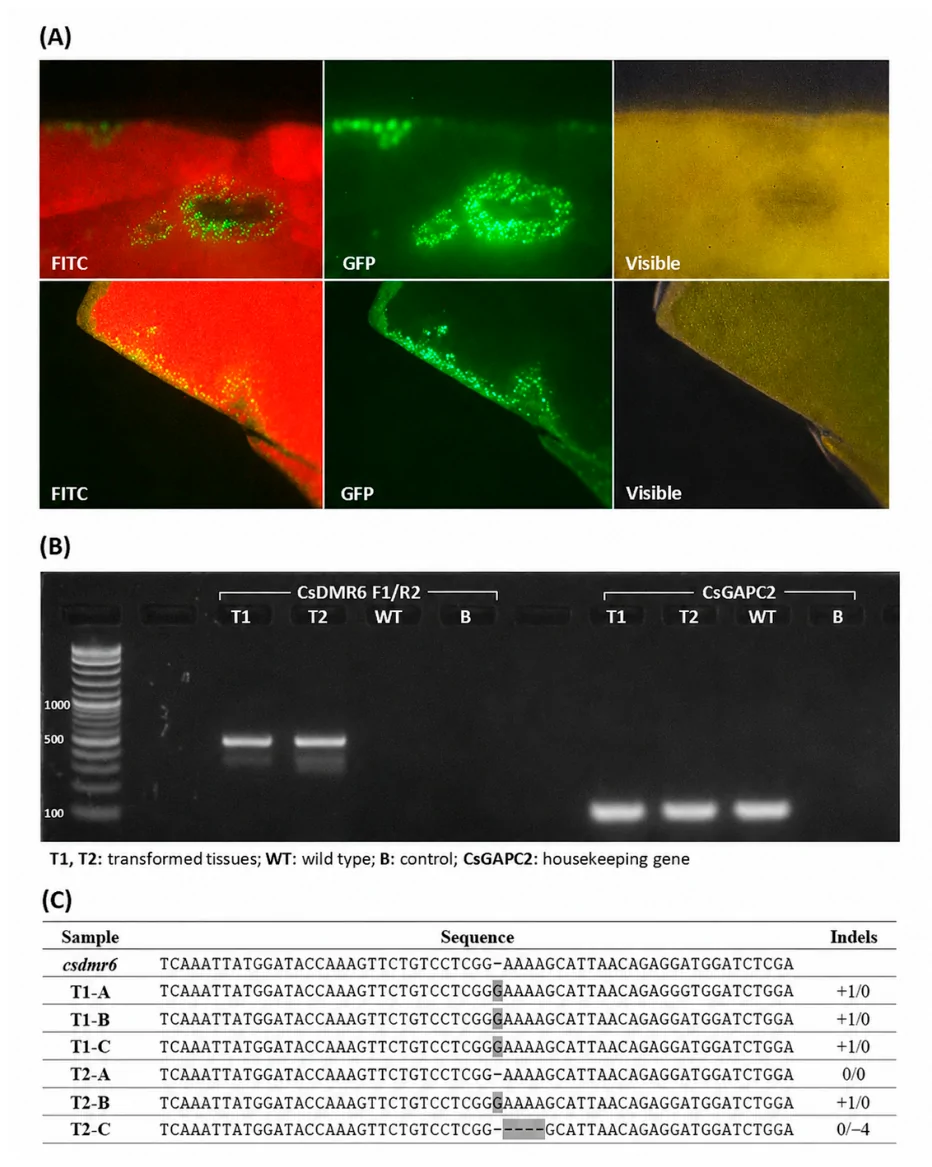
Experimental validation of dual-gRNA CRISPR/Cas9 editing of *CsDMR6* in citrus leaf tissues. (**A**) GFP fluorescence in mesophyll and vascular tissues at 6 days post-inoculation (dpi) confirms successful delivery and expression of the pVK:CsDMR6A construct. (**B**) PCR amplification using primers flanking the target region produces a diagnostic ~447 bp fragment corresponding to the expected 5.8 kb deletion relative to the ~6.2 kb wild-type allele. (**C**) Sanger sequencing of the edited fragment verifies precise junction formation between the two gRNA cut sites and reveals additional small indels at individual target sites, demonstrating efficient dual-site Cas9 activity. *CsGAPC2* reference gene (*Glyceraldehyde-3-phosphate dehydrogenase*, *cytosolic 2*) [19]. Highlighted residues indicate nucleotide positions involved in Cas9 cleavage and indel formation.

Transient transformation with *Agrobacterium* carrying pVK:CsDMR6A resulted in clear GFP fluorescence at 6 days post-inoculation (Figure 3A). Strong GFP expression occurred only in Transformation 2 (OD_600_ = 1.0; 4-day co-cultivation), whereas Transformation 1 (OD_600_ = 0.3; 2-day co-cultivation) produced fluorescence indistinguishable from autofluorescence. A total of 150–200 leaf segments were infiltrated per experiment, using elongated shoots from genotypes CsH2 and CsH3 obtained from the regeneration workflow described in Section 2.1 and Section 2.2. Three independent infiltration batches were performed to ensure reproducibility. Transformation efficiency, estimated as the percentage of leaf segments showing GFP fluorescence at 72 h post-infiltration, ranged from 18 to 25% across batches.

PCR using primers CsDMR6-F1 and CsDMR6-R2 produced a diagnostic ~447 bp fragment in both transformations (Figure 3B). This fragment corresponds to the edited allele generated by dual-gRNA cleavage, whereas the wild-type allele produces a ~6.2 kb amplicon that cannot be amplified under standard PCR conditions. T1 generated a +1 nt insertion, whereas T2 produced multiple editing events, including the complete ~5.8 kb deletion and additional small indels. Between 6 and 10 clones were sequenced per transformation condition, and deletions ranged from 350 to 420 bp at the junction, consistent with precise re-ligation between the two gRNA cut sites. Sequencing confirmed precise junction formation between the two gRNA cut sites (Figure 3C).

Agroinfiltration parameters and transient editing outcomes for the two transformation conditions evaluated are summarized in Table 2. Higher bacterial density and extended co-cultivation (T2) resulted in clear GFP fluorescence and multiple editing events, including the complete ~5.8 kb deletion. Because T1 and T2 differed simultaneously in OD_600_ and co-cultivation duration, they represent exploratory transformation environments rather than factorial comparisons; therefore, differences between them reflect optimization attempts rather than controlled variable isolation.

### 2.5. Callogenesis Characterization Under Dark Conditions

Callus induction was influenced by genotype and light conditions (Supplementary Table S1). In CiCM medium, callogenesis occurred more rapidly and at higher frequencies in complete darkness than under a photoperiod (Figure 1B). ‘Valencia’ reached 12.2% callogenesis in darkness, while CsH1 achieved 100% from leaf explants. CsH2 and CsH3 also produced callus under darkness, although at lower frequencies (18–25%) and with slower tissue expansion, consistent with their reduced vigor during shoot morphogenesis. Callus formation typically began after 10–14 days in darkness, whereas explants under photoperiod required >20 days and frequently failed to initiate callus. Although quantified, callogenesis was not used in the primary shoot-based regeneration workflow and is presented only as complementary characterization of genotype behavior. These observations confirm that callogenesis is strongly genotype-dependent and light-responsive, but does not contribute to the shoot-based regeneration pipeline optimized for CRISPR workflows.

## 3. Discussion

### 3.1. Overcoming Recalcitrance in Citrus Regeneration

The optimized regeneration workflow directly addresses the practical limitations that have constrained genome editing in *C. sinensis* [18]. Rather than reiterating the well-known recalcitrance of citrus, our results demonstrate how specific physiological bottlenecks can be resolved experimentally. Supplementation of CiSM medium with 1 mg L^−1^ GA3 effectively corrected the compact, stunted phenotype observed during shoot proliferation, enabling consistent internode elongation across genotypes. This response aligns with the established role of gibberellins in promoting shoot extension in woody perennials [20,21,22,23], but here it is shown to be essential for producing explants competent for downstream transformation in sweet orange. The genotype-specific responses observed—particularly the overstimulation phenotype in CsH3—underscore the importance of fine-tuning growth regulator concentrations when adapting regeneration protocols to hybrid backgrounds.

Callogenesis assays further revealed strong genotype-dependent responsiveness under dark conditions, particularly in CsH1, consistent with previous observations in citrus relatives [22]. Although callus formation was not incorporated into the editing workflow, these results provide complementary insight into genotype behavior and may inform future alternative regeneration routes. The differential callogenic performance of CsH2 and CsH3, which produced slower and less abundant callus, reinforces the notion that hybrid vigor does not necessarily translate into uniform in vitro responsiveness, and highlights the need for genotype-tailored regeneration strategies.

The auxin pulse strategy proved critical for resolving the second major bottleneck: rooting. Hybrid genotypes, which failed entirely under hormone-free conditions, reached 52.9% rooting following the 48 h NAA pulse. This mirrors auxin-pulse responses reported in other woody species [21,22,23] and highlights leaf maturity—specifically larger leaf surface area—as a physiological marker of rooting competence. The observation that shoots with greater leaf surface area consistently outperformed smaller shoots suggests that morphological indicators can be used prospectively to select explants with higher rooting potential, improving workflow efficiency. Together, these improvements establish a regeneration platform that is not only reproducible but also genotype-compatible, strengthening the foundation for downstream genome-editing applications in citrus. By resolving elongation, rooting, and genotype-specific physiological constraints, this workflow provides a practical route for integrating CRISPR-based editing into sweet orange improvement pipelines.

### 3.2. Strategic Target: CsDMR6 as a Susceptibility Gene

Transcriptomic evidence has shown that *CsDMR6* is strongly induced during HLB infection [7], and its role as a negative regulator of immunity is well established in *Arabidopsis* [24]. Our sequencing analysis confirmed complete conservation of both gRNA sites and PAM motifs across all evaluated sweet orange and hybrid genotypes, reinforcing *CsDMR6* as a robust and broadly applicable target. *DMR6* encodes a 2-oxoglutarate [Fe(II)]-dependent oxygenase that hydroxylates salicylic acid (SA), thereby reducing its cellular accumulation and suppressing *NPR1*-mediated immune activation [24]. Therefore, deletion of *CsDMR6* is expected to elevate SA levels, enhance basal immunity, and potentiate *NPR1*-dependent transcriptional reprogramming. This mechanistic framework explains why *DMR6* knockout confers resistance to citrus canker and supports the hypothesis that large-fragment deletion of *CsDMR6* may also reduce susceptibility to *Candidatus Liberibacter* spp. without detrimental effects on growth. The strong induction of *CsDMR6* during HLB infection, together with its conserved sequence architecture across diverse citrus backgrounds, suggests that this susceptibility node is actively exploited by the pathogen to attenuate host defense, making it an attractive target for host-directed resistance strategies.

This conservation is particularly relevant given the demonstrated functional role of *DMR6* orthologs in disease susceptibility across multiple species, including tomato [25], potato [26], and grapevine [27]. The fact that *CsDMR6* editing has already conferred resistance to citrus canker [8] further validates its suitability as a strategic node for host-directed resistance. Our results extend this evidence by demonstrating that large-fragment deletion of *CsDMR6* is feasible in sweet orange using a dual-gRNA strategy, providing a molecular configuration likely to produce complete loss-of-function alleles. These findings position *CsDMR6* as a central susceptibility hub whose removal may simultaneously enhance basal immunity and reduce vulnerability to multiple pathogens, supporting its prioritization in future genome-editing pipelines for citrus improvement.

### 3.3. Efficiency of the Dual-gRNA Geminivirus Approach and Implications for Non-Transgenic Disease Resistance

The dual-gRNA design was intended to generate a large ~5.8 kb deletion spanning most of the *CsDMR6* coding region, thereby simplifying molecular screening and increasing the likelihood of full gene knockout. The diagnostic ~447 bp edited fragment sharply contrasted with the ~6.2 kb wild-type allele, providing an unambiguous molecular signature consistent with large-deletion strategies previously applied in citrus and other crops [15,16,17]. Our transient assays revealed clear differences between the two agroinfiltration conditions. Transformation 2 (OD_600_ = 1.0; 4-day co-cultivation) produced strong GFP fluorescence and multiple editing outcomes, including the complete deletion and additional small indels, whereas Transformation 1 generated only a +1 nt insertion and lacked a detectable GFP signal. These results highlight the importance of bacterial density and co-cultivation duration for maximizing transient expression and Cas9 activity, consistent with established citrus transformation protocols [17]. The contrasting outcomes between T1 and T2 underscore the sensitivity of citrus tissues to agroinfiltration parameters and demonstrate that even modest adjustments in inoculum density and co-cultivation time can dramatically influence replicon activity and editing efficiency.

The geminivirus-based vector was central to this efficiency. Replicon systems maintain high intracellular copy numbers of CRISPR components, enhancing editing frequency without requiring stable genomic integration [15,16,28]. This transient delivery approach aligns with recent advances in transgene-free citrus editing [29,30], demonstrating that large deletions can be efficiently generated without producing stable transgenic lines. Because loss-of-susceptibility alleles do not rely on foreign gene insertion, the strategy provides a practical path toward non-transgenic citrus germplasm that remains genetically identical to elite cultivars except for the targeted deletion. This is particularly relevant for sweet orange, where consumer and regulatory acceptance strongly favor edited but non-transgenic plants for HLB management. Recent regulatory advances further strengthen the relevance of targeting *CsDMR6* for host-directed resistance. In 2024–2025, USDA–APHIS and EPA approved the first non-transgenic citrus lines carrying CRISPR-induced deletions in *DMR6*, including ‘Valencia Super’ UF W2 and ‘Hamlin Super’ UF W4, demonstrating that large-fragment edits in susceptibility genes are compatible with commercial deployment [31]. These approvals confirm that *DMR6* knockout can be achieved without stable transgene integration and that edited plants remain genetically identical to elite cultivars except for the targeted deletion. This regulatory precedent directly supports the dual-gRNA strategy used here and highlights its potential for generating commercially viable, non-transgenic germplasm for HLB mitigation. This is particularly relevant for host-directed HLB management [3,4], where regulatory and consumer acceptance strongly favor edited but non-transgenic plants.

Although this study focused on transient validation, the optimized regeneration and rooting conditions provide the necessary framework for recovering stable edited lines. Future work will integrate the validated pVK:CsDMR6A system with the stable regeneration pipeline to evaluate whether *CsDMR6* loss-of-function alleles reduce susceptibility to *Candidatus Liberibacter* infection without compromising agronomic performance. The combined evidence indicates that dual-gRNA replicon-mediated editing represents a feasible and regulatory-aligned route for producing non-transgenic, disease-resistant citrus cultivars.

### 3.4. Limitations and Future Directions

The primary limitation of this study is the absence of fully regenerated, stable edited plants. While transient assays provide strong molecular evidence of efficient dual-gRNA activity, functional validation under HLB pressure requires complete regeneration and acclimatization of edited lines. The optimized regeneration and rooting conditions developed here address the major physiological bottlenecks that previously limited stable recovery, but full integration of the dual-gRNA system into the stable transformation workflow remains an essential next step. In addition to the dual-gRNA design, potential off-target effects were evaluated using the same workflow previously applied in grapevine [15] and potato [16], combining genome browser inspection, CRISPR search tool analysis, and in silico prediction of candidate off-target loci. This pipeline screens for sequence similarity across the citrus genome and prioritizes sites with canonical PAMs and minimal mismatch profiles. Although transient assays inherently limit the recovery of complete off-target datasets, this preliminary evaluation did not identify high-risk off-target candidates for either gRNA. Future work will extend this analysis to regenerated plants through targeted sequencing of predicted loci, enabling confirmation of editing specificity and ensuring that no unintended genomic alterations compromise agronomic performance.

Additionally, although large deletions minimize the risk of partial loss of gene function, future work should evaluate potential off-target effects and confirm that *CsDMR6* knockout does not negatively impact growth or fruit quality. Given the central role of salicylic acid signaling in defense and development, phenotypic evaluation of edited lines—including growth rate, canopy architecture, and fruit set—will be essential to ensure that enhanced immunity does not incur physiological trade-offs.

Overall, the integrated workflow presented here establishes a complete and experimentally validated platform for genome editing in *Citrus sinensis*, combining optimized regeneration, reliable rooting, and efficient dual-gRNA deletion of *CsDMR6*. By resolving key physiological bottlenecks and demonstrating precise large-fragment editing, this study provides the technical foundation required to recover stable, non-transgenic edited plants suitable for functional evaluation under HLB pressure. The regulatory advances recognizing CRISPR-edited citrus lines as non-GMO further underscore the practical relevance of this approach and position susceptibility-gene editing as a realistic path toward commercial deployment. These regulatory precedents—particularly the approval of non-transgenic *DMR6*-edited citrus lines by USDA–APHIS and EPA—demonstrate that large-fragment deletions in susceptibility genes are compatible with commercial release and provide a clear pathway for future edited sweet orange cultivars. As stable edited lines are recovered and evaluated, the *CsDMR6* knockout strategy may contribute to the development of resilient citrus germplasm capable of mitigating HLB impact while preserving the genetic identity of elite cultivars. Together, these results strengthen the biotechnological capacity to respond to devastating phytosanitary threats and advance citrus improvement toward durable, host-directed disease resistance.

## 4. Materials and Methods

### 4.1. Plant Material and Aseptic Establishment

Experiments were conducted using sweet orange (*Citrus sinensis* L. Osbeck) cv. Valencia and three commercial hybrid genotypes designated CsH1, CsH2, and CsH3. For seed-derived explants, pulp and mucilage were removed by washing, followed by surface sterilization in 76% ethanol for 1 min and 20% sodium hypochlorite supplemented with Tween 20 (Sigma-Aldrich, St. Louis, MO, USA) for 20 min [32]. Seeds were rinsed five times with sterile distilled water, seed coats were removed, and embryos were germinated on CiSM1.0 medium. Embryos were placed with the radicle oriented downward to ensure consistent germination and early seedling vigor.

Juvenile internodal segments from greenhouse plants were pretreated in a fungicide solution containing 0.5 g L^−1^ Benlate (Ningbo Generic Chemical Co., Ltd., Ningbo, China) and 1.5 g L^−1^ Captan (Arysta LifeScience, Tokyo, Japan) for 1 h under agitation, followed by the same ethanol and hypochlorite sterilization protocol. Explants were trimmed to 0.8–1.2 cm to standardize tissue size across genotypes and minimize variability in regeneration responses. All cultures were maintained at 26 ± 0.5 °C under a 16 h light and 8 h dark photoperiod. Light intensity was kept at 40–50 µmol m^−2^ s^−1^ using cool-white fluorescent lamps to avoid photoinhibition during early regeneration. All cultures were maintained at 26 ± 0.5 °C under a 16 h light and 8 h dark photoperiod.

### 4.2. Culture Media and Regeneration Optimization

All media were prepared using Murashige and Skoog (MS) salts [33], 30 g L^−1^ sucrose, and 8 g L^−1^ agar, with pH adjusted to 5.7 ± 0.1. Three Citrus Shoot Multiplication (CiSM) formulations were evaluated: CiSM1.0 (1.0 mg L^−1^ BAP (Sigma-Aldrich)), CiSM1.25 (1.0 mg L^−1^ BAP and 0.25 mg L^−1^ NAA (Sigma-Aldrich)), and CiSM2.1+KIN (2.0 mg L^−1^ BAP, 0.1 mg L^−1^ NAA, and 1.0 mg L^−1^ kinetin (Sigma-Aldrich)) (Supplementary Table S2). To overcome the lack of internode elongation observed in CiSM1.0, the medium was supplemented with 1.0 mg L^−1^ GA3 (Sigma-Aldrich), a strategy previously shown to improve shoot elongation in woody perennials [34,35]. Improved gas exchange using ventilated culture jars further enhanced shoot vigor. All media were dispensed in 25 mL aliquots per vessel to standardize nutrient availability and minimize variability in shoot proliferation across genotypes.

Callogenesis was induced using Citrus Callus Induction Medium (CiCM), consisting of MS salts, 0.5 g L^−1^ MES (Sigma-Aldrich), and a hormonal combination of 3.0 mg L^−1^ BAP, 0.1 mg L^−1^ NAA, and 1.0 mg L^−1^ 2,4-D (Sigma-Aldrich) [22,36]. Comparative assays showed that complete darkness for 30 days increased the formation of friable, cream colored calli relative to photoperiod conditions. Explants were placed abaxial-side down to maximize contact with the medium and promote uniform callus initiation. Because callogenesis was not required for the regeneration-to-editing workflow, these assays were performed only to characterize genotype responsiveness. Callus tissues were not subcultured to avoid unintended somaclonal variation and to maintain consistency with the shoot-based regeneration pipeline.

### 4.3. Rooting Strategy: Auxin Pulse Treatment

To promote rhizogenesis in recalcitrant genotypes, a 48 h auxin pulse was applied following the protocol of Pandey and Tamta [21]. Elongated shoots of at least 2 cm were immersed in liquid half-strength MS (MS½) medium supplemented with 100 µM NAA and maintained in complete darkness. Shoots were fully submerged to ensure uniform auxin exposure across basal tissues, and vessels were sealed to prevent evaporation and concentration drift. After 48 h, shoots were transferred to hormone-free MS½ solid medium and returned to light conditions. Auxin pulse treatments have been shown to enhance rooting efficiency in woody perennials and citrus relatives [37,38]. Only shoots with expanded leaves and visible internode elongation were selected for pulse treatment, as preliminary assays indicated that leaf maturity strongly influenced rooting competence. Root primordia were monitored every 5–7 days, and shoots were acclimatized under high humidity once roots reached ≥1 cm.

### 4.4. In Silico Design of gRNAs and Target Validation

The *CsDMR6* locus spans approximately 6.3 kb and contains four exons. Two guide RNAs were designed using the local Biotools CRISPR Search platform (www.fruit-tree-genomics.com (accessed on 25 August 2026); tab biotools) to target conserved regions at opposite ends of the gene: gRNA2.1 in Exon 1 and gRNA1.1 in Exon 4. This configuration was predicted to generate a 5814 bp deletion. Dual-gRNA deletion strategies have been successfully applied in citrus and other crops to generate large, easily detectable deletions [15,16,17]. Target site conservation was validated across all genotypes by PCR amplification and Sanger sequencing (Supplementary Table S3). Both gRNAs were evaluated for GC content, predicted on-target efficiency, and absence of off-target sites with canonical NGG PAMs using CRISPOR and the citrus genome browser, ensuring compatibility with Cas9 activity and minimizing off-target risk. Additional gRNA modules evaluated during the design phase are included in Supplementary Table S4. Only gRNAs meeting all criteria (high predicted efficiency, conserved target sequence, and no high-risk off-targets) were selected for construct assembly.

### 4.5. Vector Assembly and Transformation

The dual-gRNA cassette was assembled into the pVK-U vector (Addgene #252744) using Golden Gate cloning [39]. The resulting construct, pVK:CsDMR6A, was introduced into *Agrobacterium tumefaciens* strain EHA105 by electroporation [14]. Positive colonies were confirmed by colony PCR using primers flanking the dual-gRNA module to ensure correct assembly prior to infiltration. Leaf segments were infiltrated with a bacterial suspension at OD_600_ = 1.0 and co-cultivated on CiCM medium supplemented with 100 µM acetosyringone (Sigma-Aldrich) for 4 days in darkness, following established citrus transformation protocols [17]. Bacterial cultures were prepared in induction medium containing 10 mM MES and 10 mM MgCl_2_ to enhance vir gene activation, and acetosyringone was added immediately before infiltration to maintain activity. Explants were gently blotted on sterile paper after infiltration to remove excess inoculum and reduce tissue necrosis during co-cultivation.

### 4.6. Molecular Detection of Genome Editing

Genomic DNA was extracted from GFP-positive tissues using the CTAB-adapted method by Acha et al. [16]. Editing was assessed by PCR using flanking primers CsDMR6-F1 and CsDMR6-R2. A diagnostic ~447 bp fragment indicated the presence of the expected 5.8 kb deletion. PCR products were cloned and sequenced to verify junction formation and characterize repair outcomes. Amplicons corresponding to non-deleted alleles (~6.2 kb) were not amplified under standard PCR conditions, providing an inherent negative selection for edited fragments and simplifying downstream screening. Sequencing chromatograms were inspected manually to confirm clean junction boundaries and to identify small indels generated by individual gRNA activity. The complete list of primers and gRNAs used for *CsDMR6* targeting and validation is provided in Supplementary Table S5. All PCR reactions were performed using high-fidelity polymerase to ensure accurate detection of deletion junctions and minimize amplification artifacts.

### 4.7. Statistical Analysis

Regeneration, elongation, rooting, and callogenesis efficiencies were treated as binomial response variables and expressed as the proportion of explants that responded relative to the total number evaluated in each condition (*n* ≥ 30 per treatment for ‘Valencia’; n = 17 for hybrid CsH1 rooting). Descriptive statistics were used to summarize these performance metrics. For each proportion, 95% confidence intervals were calculated using the Wilson method [40], implemented in GraphPad Prism (version 10; GraphPad Software, Boston, MA, USA). No additional inferential statistical tests were applied. This analytical workflow follows standard practice in citrus tissue culture research, where efficiency values are interpreted as indicators of protocol performance. All proportions were calculated independently for each genotype and treatment to avoid pseudoreplication, and confidence intervals were used solely to describe precision rather than to support hypothesis testing. Because the study focused on protocol optimization rather than comparative inference, no pairwise statistical tests were conducted.

## 5. Conclusions

We demonstrate that a dual-gRNA CRISPR/Cas9 strategy can efficiently generate a large ~5.8 kb deletion in *CsDMR6* in sweet orange, providing clear molecular evidence of precise junction formation and robust Cas9 activity. The optimized regeneration workflow—characterized by improved shoot elongation, genotype-responsive rooting, and reliable transient transformation—establishes the experimental foundation required to recover stable edited lines. Together, these results validate *CsDMR6* as a broadly conserved susceptibility gene target and provide a practical, genotype-compatible platform for host-directed genome editing in *Citrus sinensis*. By resolving key physiological bottlenecks and demonstrating efficient large-fragment deletion, this study positions susceptibility-gene editing as a feasible and scalable strategy for developing disease-resistant citrus germplasm. Future work will integrate the transiently validated pVK:CsDMR6A system with stable transformation pipelines to obtain fully edited plants and determine whether *CsDMR6* loss-of-function alleles reduce susceptibility to HLB without compromising agronomic performance. As regulatory frameworks increasingly recognize CRISPR-edited, non-transgenic citrus lines, the *CsDMR6* knockout approach may contribute to commercially viable, durable solutions for HLB management while preserving the genetic identity of elite cultivars.

## Figures and Tables

**Table 1:** Regeneration and rooting frequencies by genotype and protocol ^1^.

Genotype	Regeneration Frequency (%)	Nº of Shoots per Explant	Rooting Protocol	Rooting Efficiency (%)
Valencia	65	2.3	No pulse (Hormone-free)	30.3
			48 h NAA Pulse (100 µM)	37.1
Hybrid CsH1	52	1.7	No pulse (Hormone-free)	0.0
			48 h NAA Pulse (100 µM)	52.9

^1^ Regeneration and rooting performance across the evaluated genotypes under the optimized culture system. Regeneration was performed on CiSM medium supplemented with 1 mg L^−1^ GA3 to ensure internode elongation. Rooting data corresponds to elongated shoots with larger leaf surface area, which exhibited significantly higher responsiveness.

**Table 2:** Agroinfiltration parameters and transient editing results.

Parameter/Result	Transformation 1 (T1)	Transformation 2 (T2)
Bacterial Density (OD_600_)	0.3	1.0
Co-cultivation Period	2 days	4 days
GFP Detection (6–8 dpi)	Indistinguishable from autofluorescence	Clear fluorescence (mesophyll/vascular)
PCR Evidence (~447 bp)	Detected	Detected
Editing Events (Indels)	+1 nt insertion	~5.8 kb deletion (Complete)
		−4 nt deletion
		+1 nt insertion

## Data Availability

No new datasets were generated or analyzed in this study. All data supporting the findings are included within the article and its Supplementary Materials.

## Supplementary Materials

The following supporting information can be downloaded at: https://www.mdpi.com/article/10.3390/plants15172664/s1, Table S1: Detailed Callogenesis efficiency by light condition and explant type; Table S2: Hormonal composition of culture and induction media; Table S3: Conservation of gRNA target sites across analyzed citrus genotypes; Table S4: Alternative gRNA modules for CsDMR6 editing; Table S5: Primers and gRNAs designed for CsDMR6 targeting and validation.

## Abbreviations

The following abbreviations are used in this manuscript:

2,4-D	2,4-Dichlorophenoxyacetic acid
BAP	6-Benzylaminopurine
Cas9	CRISPR-associated protein 9
CiCM	Citrus Callus Induction Medium
CiSM	Citrus Shoot Multiplication Medium
CRISPR	Clustered Regularly Interspaced Short Palindromic Repeats
CsDMR6	*Citrus sinensis* Downy Mildew Resistance 6 gene
CsGAPC2	*Citrus sinensis glyceraldehyde-3-phosphate dehydrogenase*, *cytosolic 2*
CTAB	Cetyltrimethylammonium bromide
dpi	Days post-inoculation
GA3	Gibberellic acid
gRNA	Guide RNA
HLB	Huanglongbing (citrus greening disease)
KIN	Kinetin
MS	Murashige and Skoog medium
MS½	Half-strength Murashige and Skoog medium
NAA	1-Naphthaleneacetic acid
OD_600_	Optical density at 600 nm
PAM	Protospacer Adjacent Motif
PCR	Polymerase Chain Reaction
Sanger	Sanger dideoxy sequencing method
WT	Wild type
