# Supplementary material for: Dual-gRNA CRISPR/Cas9 Deletion of CsDMR6 in Sweet Orange Supported by Improved In Vitro Regeneration
Source: Plants (Basel). 2026 Aug 31;15(17):2664. doi: 10.3390/plants15172664 (PMC13567490; doi:10.3390/plants15172664)
Supplement: Supplementary file 1 [file plants-15-02664-s001.zip › plants-4485471-supplementary.pdf]

Supplementary Table S1. Detailed Callogenesis efficiency by light condition and explant type

Extended data showing the significant effect of darkness on callus induction.

Cultivar	Photoperiod	Explant Type	Explants (n)	Callus Induction (%)
**'Valencia'**	Light (16/8h)	Leaf	53	3.8%
	Light (16/8h)	Nodal Segment	15	13.3%
	Darkness	Leaf	49	12.2%
	Darkness	Nodal Segment	13	38.5%
**Hybrid CsH1**	Light (16/8h)	Leaf	82	4.9%
	Light (16/8h)	Nodal Segment	16	37.5%
	Darkness	Leaf	33	100%
	Darkness	Nodal Segment	31	16.1%

Supplementary Table S2. Hormonal composition of culture and induction media

Detailed concentrations of plant growth regulators used for the various stages of the platform.

Medium Type	BAP (mg/L)	NAA (mg/L)	2,4-D (mg/L)	Kinetin (mg/L)	GA_3 (mg/L)
**CiSM 1.0**	1.0	-	-	-	-
**CiSM 1.25**	1.0	0.25	-	-	-
**CiSM 2.1 + KN**	2.0	0.1	-	1.0	-
**CiSM + GA_3**	1.0	-	-	-	1.0
**CiCM (Callus)**	3.0	0.1	1.0	-	-

Supplementary Table S3. Conservation of gRNA target sites across analyzed citrus genotypes

Validation of the "universal" applicability of the editing platform.

Genotype	Target Site (gRNA 1.1)	Target Site (gRNA 2.1)	PAM Presence	Identity (%)
**'Valencia'**	Conserved	Conserved	Yes	100%
**Hybrid CsH1**	Conserved	Conserved	Yes	100%
**Hybrid CsH2**	Conserved	Conserved	Yes	100%
**Hybrid CsH3**	Conserved	Conserved	Yes	100%
**Hybrid Lemon**	Conserved	Conserved	Yes	100%

Supplementary Table S4. Alternative gRNA modules for *CsDMR6* editing

Alternative designs explored during the bioinformatic phase of the project.

Module	sgRNA Name	Sequence (5’->3’)	Predicted Deletion
**Module A (Selected)**	sgRNA 1.1	CCTCTGTTAATGCTTTTGGA	5,814 bp
	sgRNA 2.1	GTTTGTGTACCGGATTCCCG	
**Module B**	sgRNA 1.3	AGTTCTCAAAGACGACAAAT	5,376 bp
	sgRNA 2.6	AATCCGGTACACAAACTTGC	

Supplementary Table S5. Primers and gRNAs designed for *CsDMR6* targeting and validation

This table details the specific molecular tools used for the dual-gRNA CRISPR/Cas9 strategy and the subsequent validation of editing events in *Citrus sinensis*.

Element	Name	Sequence (5’->3’)	Predicted Size / Deletion
**gRNAs**	gRNA 1.1	CCTCTGTTAATGCTTTTGGA	5,814 bp (Deletion)
	gRNA 2.1	GTTTGTGTACCGGATTCCCG	
**Primers**	CsDMR6-F1	TTCCTAGAAGGTGCAAATACTTTTG	623 bp (WT)
	CsDMR6-R1	AAGGTAGATGGATTTGAAGGCC	
	CsDMR6-F2	TCGGCTATAGAGTGCTGGAATT	981 bp (WT)
	CsDMR6-R2	TCAGATACATCACTACTCAAAGGTT	
	CsGAPC2-F	TCTTGCCTGCTTTGAATGGA	80 bp (Housekeeping control)
	CsGAPC2-R	TGTGAGGTCAACCACTGCGACAT	
